# Efficacy and safety of autologous adipose-derived stromal vascular fraction enriched with platelet-rich plasma in flap repair of transsphincteric cryptoglandular fistulas

**DOI:** 10.1007/s10151-021-02524-6

**Published:** 2021-10-04

**Authors:** W. R. Schouten, J. H. C. Arkenbosch, C. J. van der Woude, A. C. de Vries, H. P. Stevens, G. M. Fuhler, R. S. Dwarkasing, O. van Ruler, E. J. R. de Graaf

**Affiliations:** 1 Department of Surgery, IJsselland Hospital, Prins Constantijnweg 2, 2906 ZC Capelle aan den IJssel, The Netherlands; 2 Department of Gastroenterology and Hepatology, Erasmus University Medical Center, Rotterdam, The Netherlands; 3 Department of Plastic Surgery, Velthuis Clinics / PRSclinics, Rotterdam, The Netherlands; 4 Department of Radiology, Erasmus University Medical Center, Rotterdam, The Netherlands

**Keywords:** Perianal fistula, Surgery, Cryptoglandular, Stromal vascular fraction, Platelet-rich stroma, Platelet-rich plasma

## Abstract

**Background:**

Transanal advancement flap repair of transsphincteric fistulas is a sphincter-preserving procedure, which frequently fails, probably due to ongoing inflammation in the remaining fistula tract. Adipose-derived stromal vascular fraction (SVF) has immunomodulatory properties promoting wound healing and suppressing inflammation. Platelet-rich plasma (PRP) reinforces this biological effect. The aim of this study was to evaluate the efficacy and safety of autologous adipose-derived SVF enriched with PRP in flap repair of transsphincteric cryptoglandular fistulas.

**Methods:**

A prospective cohort study was conducted including consecutive patients with transsphincteric cryptoglandular fistula in a tertiary referral center. During flap repair, SVF was obtained by lipoharvesting and mechanical fractionation of adipose tissue and combined with PRP was injected around the internal opening and into the fistulous wall. Endpoints were fistula healing at clinical examination and fistula closure on postoperative magnetic resonance imaging (MRI). Adverse events were documented.

**Results:**

Forty-five patients with transsphincteric cryptoglandular fistula were included (29 males, median age 44 years [range 36–53 years]). In the total study population, primary fistula healing was observed in 38 patients (84%). Among the 42 patients with intestinal continuity at time of surgery, primary fistula healing was observed in 35 patients (84%). In one patient, the fistula recurred, resulting in a long-term healing rate of 82%. MRI, performed in 37 patients, revealed complete closure of the fistula tract in 33 (89.2%). In the other patients, the tract was almost completely obliterated by scar tissue. During follow-up, none of these patients showed clinical signs of recurrence. The postoperative course was uneventful, except for three cases; venous thromboembolism in one patient and bleeding under the flap, necessitating intervention in two patients.

**Conclusions:**

Addition of autologous SVF enriched with PRP during flap repair is feasible, safe and might improve outcomes in patients with a transsphincteric cryptoglandular fistula.

**Trial registration:**

Dutch Trial Register, Trial Number: NL8416, https://www.trialregister.nl/

## Introduction

Transanal advancement flap repair (TAFR) is one of the most important sphincter-preserving procedures for the treatment of transsphincteric cryptoglandular fistulas. Despite many attempts to improve outcome, this procedure still fails in about 40% of tertiary referral patients with complex fistula [1]. This high failure rate is probably due to ongoing inflammation in the remaining tract near the origin of the fistula [2]. Inflammation in fistula tracts exhibits characteristics of chronic inflammation and proliferation of fibroblasts and epithelial cells [3]. Although living bacteria are infrequently found in fistula tracts, remnants of microorganism like peptidoglycan are found and might initiate chronic inflammation [2, 4, 5]. Peptidoglycan has potent pro-inflammatory properties by stimulating production of interleukin-1β and other inflammatory mediators. Recently, it was demonstrated that these cytokines are expressed in biopsies taken from chronic cryptoglandular fistulas [2, 5]. These findings suggest that inflammatory mechanisms could play a role in the persistence of anal fistulas [3]. Therefore, it is theoretically possible to enhance the outcome of sphincter-preserving procedures by additional suppression of chronic inflammation.

Stromal vascular fraction (SVF), which is extracted from adipose tissue, is thought to have the potential to suppress chronic inflammation and promote tissue regeneration [6, 7]. For many years, SVF was harvested by enzymatic digestion of lipoaspirate and subsequent centrifugation. Frequently, enzymatically digested SVF was expanded through in vitro culture to obtain adipose-derived stromal cells (ADSCs). This type of processing is time-consuming, expensive, and associated with the inherent risk of contamination [8]. Moreover, it only yields a single-cell suspension without other types of regulatory cells, extracellular matrix, or microvasculature [9]. Two recent developments have significantly changed both the processing and the use of SVF: first, the introduction of mechanical fractionation, which makes enzymatic digestion with collagenase, no longer required [9]; second, the finding that freshly isolated ADSCs show better differentiation potential as compared to culture-expanded ADSCs [8, 10]. SVF, obtained by mechanical fractionation, consists of a heterogeneous cellular mixture including ASCs embedded in extracellular matrix and surrounded by an extensive network of small blood vessels. Platelet-rich plasma (PRP) is thought to reinforce the biological effect of SVF [11]. SVF enriched with PRP is also referred to as platelet-rich stroma (PRS), which may provide a unique tool to suppress chronic inflammation, and promote local tissue repair and even regeneration, as described by Stevens et al. [12].

The aim of this study was to assess the feasibility, safety, and efficacy of additional injection of autologous SVF enriched with PRP in TAFR of transsphincteric fistulas of cryptoglandular origin in tertiary referral patients.

## Materials and methods

### Study design

Between December 2017 and February 2020, consecutive patients with transsphincteric cryptoglandular fistula, who were scheduled for TAFR in a tertiary referral center, were enrolled in this prospective cohort study. Exclusion criteria were age under 18 years, associated pelvic abscess(es), rectovaginal fistula, the presence of a second internal opening above the dentate line, history of Crohn’s disease, immune compromised status, hematological disorders, coagulation disorders, and/or any oncological event in the previous 5 years. All patients underwent standardized TAFR with additional injection of autologous SVF and PRP performed by three colorectal surgeons (W.R.S, O.R., and E.G.). In patients with associated abscess(es) detected on preoperative MR imaging, drainage was performed with seton or drain placement prior to the flap procedure.

Primary endpoint was primary fistula healing at clinical examination during follow-up, defined as complete closure of the external opening(s) without any fluid discharge from the fistula tract. Secondary endpoints were the occurrence of adverse events, obliteration of the fistula tract on magnetic resonance imaging (MRI), recurrence rate after initial fistula healing, and long-term follow-up. Postoperative outpatient visits at which physical examination was performed were scheduled at 6 weeks and 3, 6, and 12 months. However, if patients had no clinical complaints and were discharged before 12 months, telephone consultation was used to obtain their clinical status. In case of uncertainty, patients were seen at the outpatient clinic for physical examination. Prior to TAFR, the patient’s medical history was recorded and physical examination was performed. Written informed consent was obtained from all subjects before entering the study, which was approved by the Medical Ethical Committee of the Erasmus Medical Center.

### MRI evaluation

Prior to surgery, MRI was performed in all patients using a 1.5 T. system with a four-channel phased array pelvic coil. Field of view consisted of the lower pelvis, perineum, and skin area with full display of the anus and lower mid rectum. The MRI protocol included T2-weighted (T2W) sequences in three planes: axial T2W with fat saturation, and sagittal and coronal T2W. Fistulas were identified based on hyperintense (white) signal. The course of the fistula tract, presence of secondary tracts, and associated abscess(es) were examined. When physical examination revealed closure of the external opening(s), MRI was repeated. Complete closure of the fistula tract(s) was defined as total absence of hyperintense signal. An experienced radiologist who was blinded for the clinical status (R.S.D.) interpreted the anonymized images of the pre- and postoperative MRI’s.

### Operative techniques

**Fig. 1:**
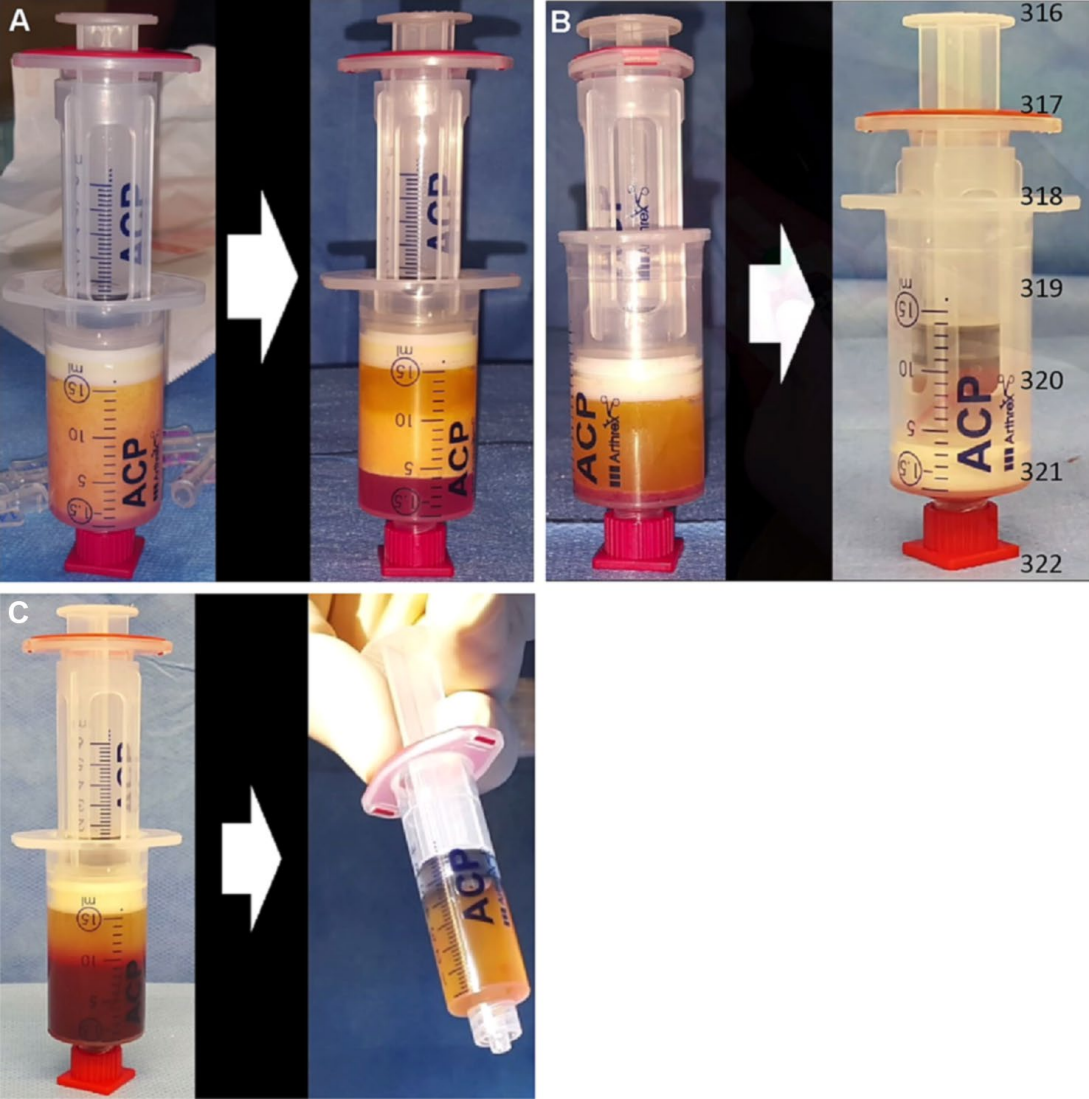
Mechanical fractionation procedure of SVF combined with PRP procedure. Lipoaspirate harvested by liposuction from subcutaneous fatty tissue is centrifuged (5 min, 2500 rpm), resulting in ± 10 ml condensed fatty tissue (**A**), mechanically fractionated and centrifuged again (5 min, 2500 rpm) to obtain 1 ml SVF (**B**). A venous blood sample (15 ml) is centrifuged (4 min, 1500 rpm) after which 4–5 ml PRP was obtained (**C**). *SVF* stromal vascular fraction; *PRP* platelet-rich plasma

After induction of general endotracheal anesthesia, metronidazole (500 mg) and cefuroxime (2000 mg) were administered intravenously. The external opening(s) of the fistula was enlarged by coring out to the exterior of the external anal sphincter. If flap procedure was considered feasible, lipoaspiration for the harvesting of SVF was initiated [13]. A video of the procedure was previously published [13]. Due to the hypothetical risk of subcutaneous infection, liposuction was always performed prior to flap repair. A small paravertebral skin incision was made bilaterally approximately 5 cm cranial to the posterior superior iliac spine. Bilaterally, the subcutaneous adipose tissue was infiltrated with 20–40 ml 0.9% saline solution, containing 20 mg/ml xylocaine and 5 µgram/ml adrenaline. Using vacuum, 15 ml of lipoaspirate was harvested bilaterally, with a double syringe (Arthrex GMBH, München, Germany). Hereafter, each double syringe was put in a sterile centrifuge bucket. During the first centrifugation cycle (5 min, 2500 rpm), the TAFR procedure was continued. A Lone Star retractor (Lone Star Retractor System, Lone Star Medical Products^®^, Inc., Houston, TX) was used to expose the internal opening, which was enlarged and the remaining crypt-bearing tissue was excised. A small rim of anoderm, distal of the internal opening, was excised to create a neo-dentate line. A flap comprising approximately one-third of the circumference, consisting of mucosa, submucosa, and some of the most superficial fibers of the internal anal sphincter, was raised from the level of the dentate line and mobilized over a distance of 4–6 cm proximally. Centrifugation of the lipoaspirate resulted in three separated fractions: oil, condensed fatty tissue, and aqueous fraction (Fig. 1A, I). The oil was transferred into the smaller inner syringe and discarded. The aqueous fraction was drained from the larger outer syringe. The remaining condensed fatty tissue was transferred to a 10 ml syringe connected to another 10 ml syringe by a 1.4 mm connector and fractionated by vigorously transferring the fatty tissue 30 times through this connector. After mechanical fractionation, the fatty tissue was centrifuged (5 min, 2500 rpm) (Fig. 1A, II). After the second centrifugation, the upper oily fraction was removed, resulting in 1 ml SVF [9]. A venous blood sample (15 ml) was centrifuged (4 min, 1500 rpm) after which 4–5 ml PRP was obtained (Fig. 1B). PRP, harvested this way, contains approximately 5 × 10^8^ platelets/ml [11]. SVF was combined with PRP, with a median volume of 7 cc, and injected with 1 cc syringes in microblebs around and through the enlarged internal and external opening into all quadrants of the fistula wall. Hereafter, the internal opening was closed with absorbable sutures 2/0 Vicryl (Ethicon, Inc., Somerville, NJ, USA). The flap was advanced and sutured to the neo-dentate line with absorbable sutures 2/0 Monocryl (Ethicon, Inc., Somerville, NJ, USA). Standard local postoperative protocol included administration of postoperative antibiotics, discharge from the hospital after 3 days if possible, and no specific regulations for bed rest.

### Statistical analysis

Continuous variables were recorded as means (standard deviation (SD)) for normally distributed data or medians (interquartile range [IQR]) for non-parametric data and categorical variables collated as frequencies and percentages. Statistical analysis was performed with IBM Statistical Packages for Social Sciences (SPSS) for Windows (lBM Corp., Armonk, NY, USA).

## Results

### Demographics

Forty-five consecutive patients (29 males [64.4%]) with a median age of 44 years [range 36–53 years]) were enrolled. All patients completed 12 months of follow-up (median length of follow-up 1.7 years [IQR 1.3–2.0]). Median duration of symptoms prior to surgery was 2.5 years (range 0–16 years). Forty-three patients (95.6%) previously underwent fistula surgery. The median number of prior procedures was 4 (IQR 2–6). In 22 patients (51.2%) prior surgical procedures concerned attempts at fistula closure (Table 1). At time of surgery, 3 patients were diverted. None of the patients had any synchronous perianal pathology.

**Table 1:** Baseline characteristics of patient with cryptoglandular fistulas who underwent SVF with PRP

Variables	*N* = 45
Age (years)	
Median (IQR)	44.0 (36.2–53.3)
Sex	
Male (%)	29 (64.4)
Follow-up (years)	
Median (IQR)	1.7(1.3–2.0)
Duration of symptoms prior to surgery (years)	
Median (IQR)	2.5 (1.1–5.5)
Range	0–15.8
Deviating stoma at time of surgery	
Number of patients (%)	3 (6.7)
Complex fistula (high transsphincteric and/or multiple side tracts)	39 (86.7)
Fistula classification	
High transsphincteric (%)	37 (82.2)
Low transsphincteric (%)	8 (17.8)
Fistula extension	
No side tracts (%)	21 (46.7)
1 side tract	11 (24.4)
≥ 2 side tracts	13 (28.9)
Prior fistula surgery	
Number of patients (%)	43 (95.6)
Total number of fistula procedures prior to PRS surgery	
Median (IQR)	4 (2–6)
Prior fistula procedures aimed at fistula repair	
TAFR and/or LIFT (%)	22 (48.9)
Fistulotomy and/or fistulectomy (%)	4 (9)
Previous abscess drainage	
Abscess drainage without drain or seton placement	28 (62.2)
Abscess drainage with drain or seton placement	14 (31.1)
Seton placement alone	28 (62.2)

*SVF* stromal vascular fraction, *PRP* platelet-rich plasma, *PRS* platelet-rich stroma, *TAFR* transanal advancement flap repair, *LIFT* ligation of intersphincteric fistula tract

### Surgical procedure

The mean operating time was 82 min (± SD 22). The median yield of SVF and PRP was 7 ml (range 3–12 ml). The creation of the flap was uneventful except for one patient in whom a longitudinal tear occurred, which could be restored. The median length of hospital stay was 3 days (range 2–4 days).

### Outcomes

#### Primary endpoint

Primary fistula healing was observed in 38/45 patients (84%). One of these patients encountered recurrence, resulting in a long-term success rate of 82% (37/45). Six patients in whom the fistula persisted or recurred underwent further treatment aimed at fistula closure. Two patients were successfully treated with subsequent laser coagulation. Four patients underwent a second TAFR with injection of SVF enriched with PRP, which was successful in one patient. The three patients with persisting fistula refrained from further treatment.

#### Secondary endpoints

**Fig. 2:**
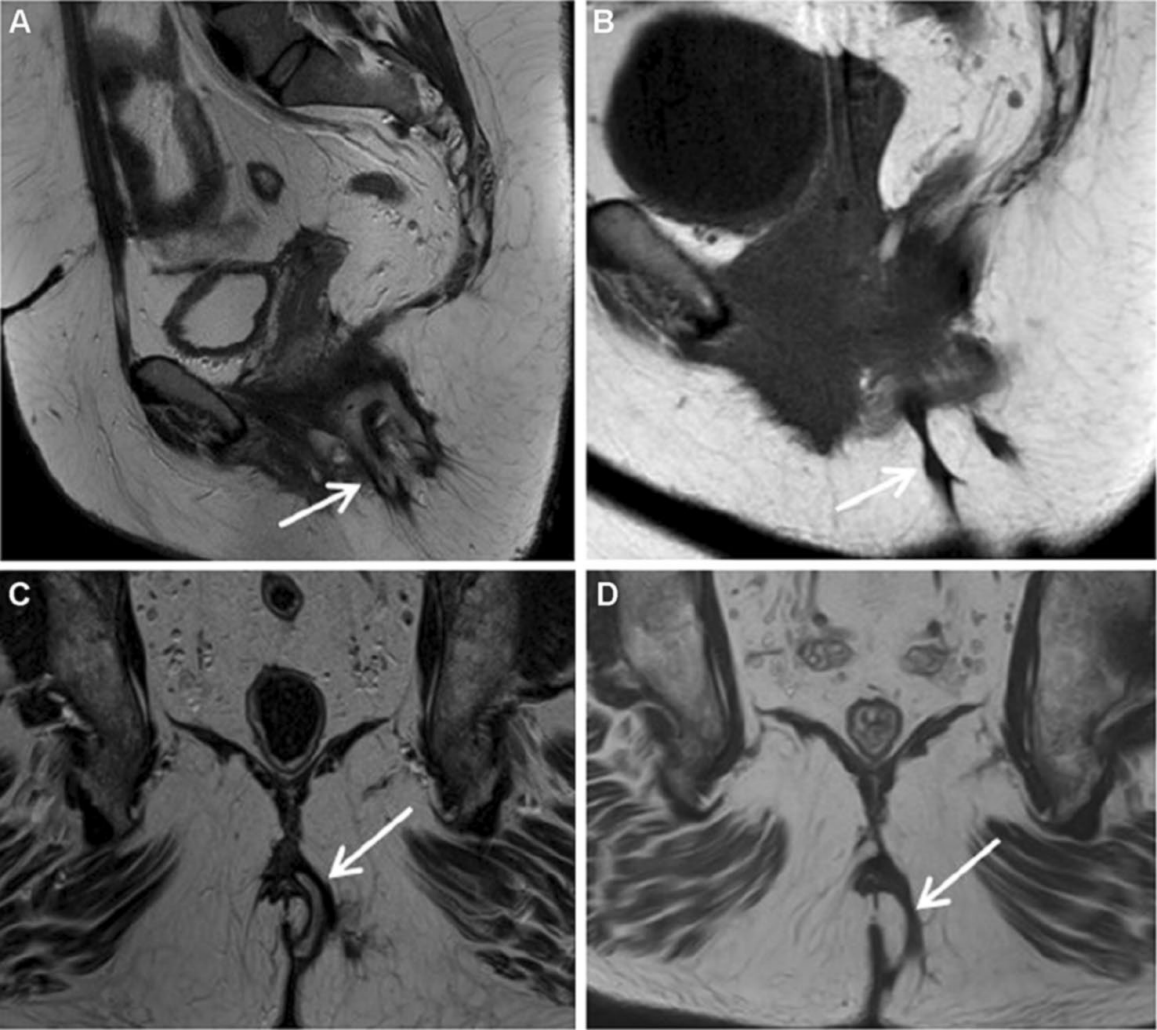
Preoperative and postoperative MRI in 2 patients with fistula healing, showing: **A** preoperative MRI (sagittal view) of a transsphincteric fistula with horseshoe extension complete obliteration of the fistula tract (patient 1); **B** postoperative MRI (sagittal view) at 6 months shows complete obliteration of the fistula tract with fibrotic tissue (patient 1); **C** preoperative MRI (coronal view) of a transsphincteric fistula (patient 2); **D** postoperative MRI (coronal view) at 6 months shows complete obliteration of the fistula tract with fibrotic tissue (patient 2). *MRI* magnetic resonance imaging

**Fig. 3:**
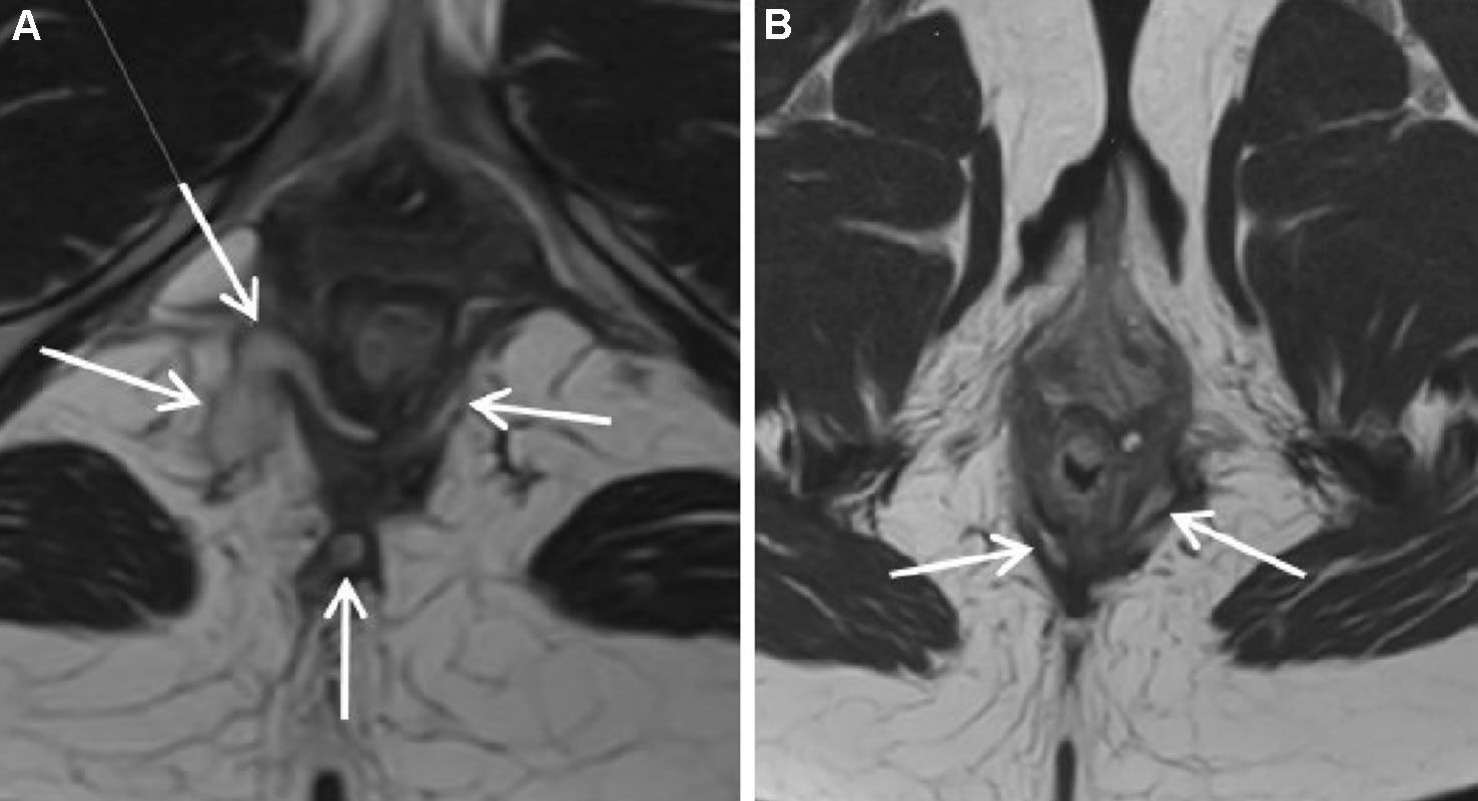
Preoperative and postoperative MRI in a patient with fistula healing, showing incomplete obliteration of the fistula tract: **A** preoperative MRI (transverse view) of a transsphincteric fistula, showing parts of the fistula tract at both lateral side and in the posterior midline; **B** postoperative MRI (transverse view) at 6 months shows incomplete obliteration of the fistula tract with remains of the fistula in the intersphincteric plane at the right and left sides. *MRI* magnetic resonance imaging

The preparation of SVF and PRP was carried out without problems. No adverse events were encountered at the lipoharvesting sites. The postoperative course was uneventful, except in three cases; venous thromboembolism in one patient and bleeding under the flap, necessitating re-intervention, in two patients. Following fistula healing, MRI evaluation was performed in all patients, except for one patient who suffered from claustrophobia. The median length of time between operation and postoperative MRI was 6.6 (IQR 4.7–7.5) months. MRI revealed complete closure in 33 out of 37 patients with clinical healing (89.2%) (Fig. 2). In two patients, the fistula tract was almost completely filled with fibrotic tissue, except for a minor part (Fig. 3). In the other two patients, the fistula tract was partly obliterated by scar tissue. To date, these four patients have not shown any signs of recurrence. Of the three patients who were diverted at time of surgery, one patient underwent restoration of bowel continuity following fistula healing. To date, the other two patients have not undergone stoma reversal despite complete fistula closure. In the 42 patients with intestinal continuity at time of surgery, primary fistula healing was observed in 35 patients (84%).

## Discussion

The present study showed that additional treatment with autologous adipose-derived SVF enriched with PRP is feasible, safe, and seems to result in a better outcome of flap repair in patients with transsphincteric, cryptoglandular fistula. The median duration of follow-up was 1.7 years, which is adequate to determine the overall healing rate [14]. Our study also revealed that MRI is a useful tool to confirm fistula healing in cryptoglandular disease.

A recent systematic review and meta-analysis comprising 721 patients revealed a weighted overall TAFR success rate of 74.6% [15]. However, the reported healing rates ranged widely from 33 to 95%, probably due to major heterogeneity with respect to the number of patients with a complex or recurrent fistula. Most reviewed series were small, with a median number of patients of only 35 [15]. Our group reported the largest series in this review in 2014, which comprised 252 patients with a healing rate of only 59% [1]. The first study conducted by our group in the early 90 s showed fistula healing after flap repair in 75% of the patients [16], suggesting that healing rates have dropped in the past decades probably inherent to increasing numbers of tertiary referrals with complex fistula. Thus, a weighted overall success rate of more than 74% does not reflect daily practice of tertiary referral centers.

A high failure rate after conventional TAFR without additional treatment is probably due to ongoing chronic inflammation in the residual fistula tissue [4]. This notion is supported by the fact that fistulectomy, the only intervention resulting in complete excision of all inflamed tissue, obtains much higher healing rates then can be obtained with any sphincter-preserving procedure. Furthermore, our group observed that in almost all patients who failed TAFR, the sutured flap was healed completely except at the original internal opening. This indicates that ischemia and flap retraction play a minor role in failure [14]. It is conceivable that the outcome of TAFR may be enhanced by suppressing chronic inflammation while simultaneously promoting wound healing. This prompted us to select SVF enriched with PRP to achieve this goal.

The use of mesenchymal stromal cells (MSCs) for regenerative medicine has been advocated since their discovery in the early 70s, due to their anti-inflammatory properties and ability to differentiate into multiple lineages [17, 18]. The easy accessibility, high vascularization, and relative high yield of ADSCs make adipose tissue a great source. While the use of isolated ADSCs has been shown to be efficacious in several wound healing models [19, 20], it often requires in vitro expansion of ADSCs. This affects their biological activity, is expensive, and may potentially introduce contamination [8, 10, 21]. Mechanically fractionated SVF may perform equally well or even better than isolated and culture-expanded ADSCs [22]. Cells present in SVF include macrophages, stromal cells, and endothelial cells, which play a pivotal role in tissue repair [23, 24]. Like ASCs, SVF has immunomodulatory properties and contributes to the downregulation of the pro-inflammatory cytokines IL-1B and IL6 [6, 25]. It also has distinct angiogenetic properties, which are more effective than those of ADSCs alone [20]. These properties may be related to the presence of intact microvasculature and extracellular matrix within SVF, as prevascularisation can enhance neovascularization [26, 27]. Furthermore, SVF improves and stabilizes the microvasculature assembled by endothelial cells [7, 28].

PRP is a rich source of growth factors and enhances proliferation, differentiation, and neovascularization induced by stromal cells [29–31]. In addition, the fibrin network of PRP has the potential to serve as a scaffold, holding cells, and platelets in a three-dimensional arrangement. This interaction may enhance and prolong survival of stromal cells at the administration site [32]. In porcine and murine models, stromal cells enriched with PRP resulted in better revascularization and wound healing as compared to stromal cells alone [33, 34]_._ This synergistic interaction prompted our decision to use SVF enriched with PRP. It should be noted that the composition of autologous preparations of SVF and PRP might differ from donor to donor, which in turn potentially affects healing rates [35].

The role of cultured stem cells in the treatment of anal fistulas, especially in Crohn’s disease, has gained increasing attention during the last decade (Table 2). Herreros et al. randomly assigned patients with transsphincteric, cryptoglandular fistula to receive 20 million cultured ADSCs (*n* = 64), 20 million ADSCs plus fibrin glue (*n* = 60), or fibrin glue alone (*n* = 59) after simple closure of the internal opening. At 1 year, observed healing rates were 57%, 52%, and 37%, respectively [36, 37]. Choi et al., also using ADSCs, observed a healing rate of 69% at 8 weeks [38]. The use of autologous SVF in the treatment of cryptoglandular fistulas was first reported in 2015. After enzymatic digestion of the lipoaspirate within a fully automated Celution 800/CRS system, it was washed and centrifuged to obtain SVF, which was injected into and around the fistula tract upon simple closure of the internal opening. At 46 months, this procedure was found to be successful in 4/7 patients [39]. Naldini and co-workers obtained SVF by mechanical fractionation of fatty tissue, similar to our fractionation technique, and showed a healing rate of 74% of 19 patients after 12 months of SVF injection into the wall of the fistula tract, followed by two-layered closure of the internal opening [40]. As primary treatment, the healing rate was 83%, whereas the success rate was 57% in patients in whom previous procedures had failed [40]. The results obtained from these studies suggest that closure of the internal opening with additional injection of stem or stromal cells results in a better outcome than closure of the internal opening alone. In 2018, Wainstein et al. used enzymatically isolated and culture-expanded ADSCs combined with PRP as an adjunct to flap repair in 9 patients with 11 fistulas due to Crohn’s disease. According to the authors, 10 fistulas healed after a median follow-up of 31 months [41]. This striking result indicates that additional injection of stromal cells combined with platelets may also be beneficial for patients with Crohn’s disease eligible for flap repair.

**Table 2:** Autologous adipose-derived stromal/stem cells (ADSCs) in cryptoglandular anal fistulas

Author	Year	Number of patients	Processing ADSCs	Surgical procedure	Follow-up (months)	Healing (%)
Herreros [36] (Phase III trial)	2012	64	Enzymatic isolation + culture expansion	Simple closure	12	57
Garcia, Herreros [37] (Phase II trial)	2009	60	Enzymatic isolation + culture expansion	Simple closure + glue	12	52
Borowski [39]	2015	7	Enzymatic isolation SVF alone	Simple closure	46	57
Choi [38]	2017	13	Enzymatic isolation + culture expansion	Simple closure	2	69
Naldini [40]	2018	19	Mechanical fractionation SVF alone	Two-layered closure	12	74
Present study	2020	27	Mechanical fractionation SVF + PRP	Flap repair	8	85

*SVF* stromal vascular fraction, *PRP* platelet-rich plasma

The main limitations of our study were the small sample size and lack of a control group. A randomized study is needed to compare the effect of SVF enriched with PRP treatment in other perianal pathologies, including Crohn’s disease, and determine whether injection of SVF enriched with PRP without flap repair is also feasible. The strength of our study lies in the new and simple perioperative technique to harvest SVF by mechanical fractionation of adipose tissue. This method was found to be quick, inexpensive, and independent of laboratory facilities, providing immediate preparation of ADSCs. Another strong feature of this study is our choice to combine SVF with PRP, based on their synergistic interaction. To the best of our knowledge, the present study is the first study that evaluates the effect of SVF enriched with PRP as an adjunct to TAFR for cryptoglandular fistulas.

## Conclusions

The long-term success rate of 82% is higher than in other studies, indicating that additional treatment with SVF enriched with PRP is a promising tool to improve the outcome of TAFR in patients with a transsphincteric, cryptoglandular fistula in a tertiary referral center. Further research is needed to define the appropriate ratio between, and optimal amount of, SVF and PRP in correlation with clinical outcome.

## Data Availability

Equipment supplied by Arthrex GMBH, München, Germany.
